# A quantitative study on Muslim milk mother’s understanding of the Islamic concept of wet nursing

**DOI:** 10.1371/journal.pone.0265592

**Published:** 2022-05-19

**Authors:** Salasiah Hanin Hamjah, Norsyamlina Che Abdul Rahim, Nurhidayah Muhammad Hashim, Norainan Bahari, Zuliza Mohd. Kusrin, Latifah Abdul Majid, Rafeah Saidon, Muhamad Zariff Illias

**Affiliations:** 1 Research Centre for Da’wah & Leadership, Faculty of Islamic Studies, Universiti Kebangsaan Malaysia, Bandar Baru Bangi, Selangor, Malaysia; 2 Centre for Nutrition Epidemiology Research, Institute for Public Health, National Institutes of Health, Ministry of Health Malaysia, Shah Alam, Selangor, Malaysia; 3 Academy of Contemporary Islamic Studies, Universiti Teknologi MARA, Shah Alam, Selangor, Malaysia; 4 Faculty of Syariah and Law, Kolej Universiti Islam Antarabangsa Selangor, Kajang, Selangor, Malaysia; 5 Research Centre for Syariah, Faculty of Islamic Studies, Universiti Kebangsaan Malaysia, Bandar Baru Bangi, Selangor, Malaysia; 6 Research Centre for al-Quran and al-Sunnah, Faculty of Islamic Studies, Universiti Kebangsaan Malaysia, Bandar Baru Bangi, Selangor, Malaysia; Shahjalal University of Science and Technology, BANGLADESH

## Abstract

**Background:**

The practice of wet nursing or breastfeeding another woman’s child in Malaysia, especially among Muslim mothers, is on the rise. This is due to the emergence of public awareness of the benefits of breast milk to children. However, it is claimed that some mothers do not have a clear understanding of the Islamic rulings concerning wet nursing, especially those related to mahram (prohibition to marry), nasab (lineage) and inheritance. Hence, the purpose of this study is to examine the level of understanding of the legal implications of wet nursing and the factors influencing the practice among Muslim mothers who have practiced breastfeeding.

**Methods:**

This was a cross-sectional descriptive study that was conducted between January and July 2019. This study was conducted with 100 Muslim mothers who had breastfed others’ child(ren) in Selangor. Data were obtained using a validated questionnaire (Cronbach’s alpha = 0.8) and processed using the Statistical Package for the Social Sciences (SPSS) software.

**Results:**

The results show that the majority of the respondents understand the basic Islamic rulings on wet nursing, especially on the persons prohibited to marry, conditions and feeding method. However, the respondents need to further understand the rulings related to *nasab* (lineage), guardianship and inheritance involving the milk child. Various factors that have influenced wet nursing in society were also found.

**Conclusions:**

This study has significant implications for the need to have more activities to create awareness and enhance the understanding related to wet nursing among Muslim women in society. Therefore, more research studies related to wet nursing and the impact of the practice should be conducted to offer better solutions to society.

## Introduction

Mother’s breast milk is scientifically proven to contain essential nutrients beneficial for the physical and mental development of infants for their growth [1, 2]. Mother’s breast milk is also found to help strengthen infants’ antibodies or immunity against dangerous diseases [3–5]. Due to awareness of the importance of mother’s milk for an infant’s healthy growth and intelligence, mothers are determined to breastfeed their infants, even though they are working mothers. The technological invention of a sophisticated milking machine helps the process of feeding breast milk as expressed milk can be frozen and given to the infant later while the mother is working [6–9]. In addition, the development of social networks such as WhatsApp, Twitter, and Facebook have allowed the formation of online support groups for mothers. Other than moral support from husbands and family, online support groups also help to motivate mothers in feeding infants breast milk exclusively for at least six months [7, 10–13].

Increasing public understanding of breast milk’s nutrients for infants causes mothers who are unable to breastfeed their infants by themselves to hire milk mothers or even buy breast milk from other women. This practice has given rise to the phenomenon of wet nursing. Wet nursing can be explained as any form of breastfeeding provided by someone who is not the infant’s biological mother [14, 15]. Wet nursing is a very old practice, especially among Muslim cultures. The Noble Quran also mentions this: ‘If they are pregnant, spend on them until they lay down their burden; then if they suckle for you, give them their recompense and enjoin one another among you to do good; and if you disagree, another (woman) shall suckle for him [Chapter 65: 6]’.

There is very famous tradition about the last prophet, Muhammad (S.A.W.), having been fed breast milk by Halimah al-Sa’adiyah (R.A.). Biographers accept that the Prophet was nursed by Thuwaybah, servant of Abu Lahab, for a while. Then, Halimah al-Sa’adiyah, daughter of Abu Dhu’ayb, accepted him into her charge because she could not find another to care for this orphan child. Halimah related that after she took Muhammad with her, she found all types of blessings and goodness. She nursed him for two whole years, and then she brought him back to his mother [16–18].

Although breastfeeding someone else’s child is permissible in Islam, women who become milk mothers need to understand the effects in Shariah law, especially if they have established milk kinship with the infants. The problem among milk mothers is that they might not know the legal effects in terms of Shariah law. If this ignorance is not overcome, it is feared that there might be future marriages between milk siblings, which contradicts the objectives of Shariah law, namely, to preserve a person’s lineage.

**Fig 1:**
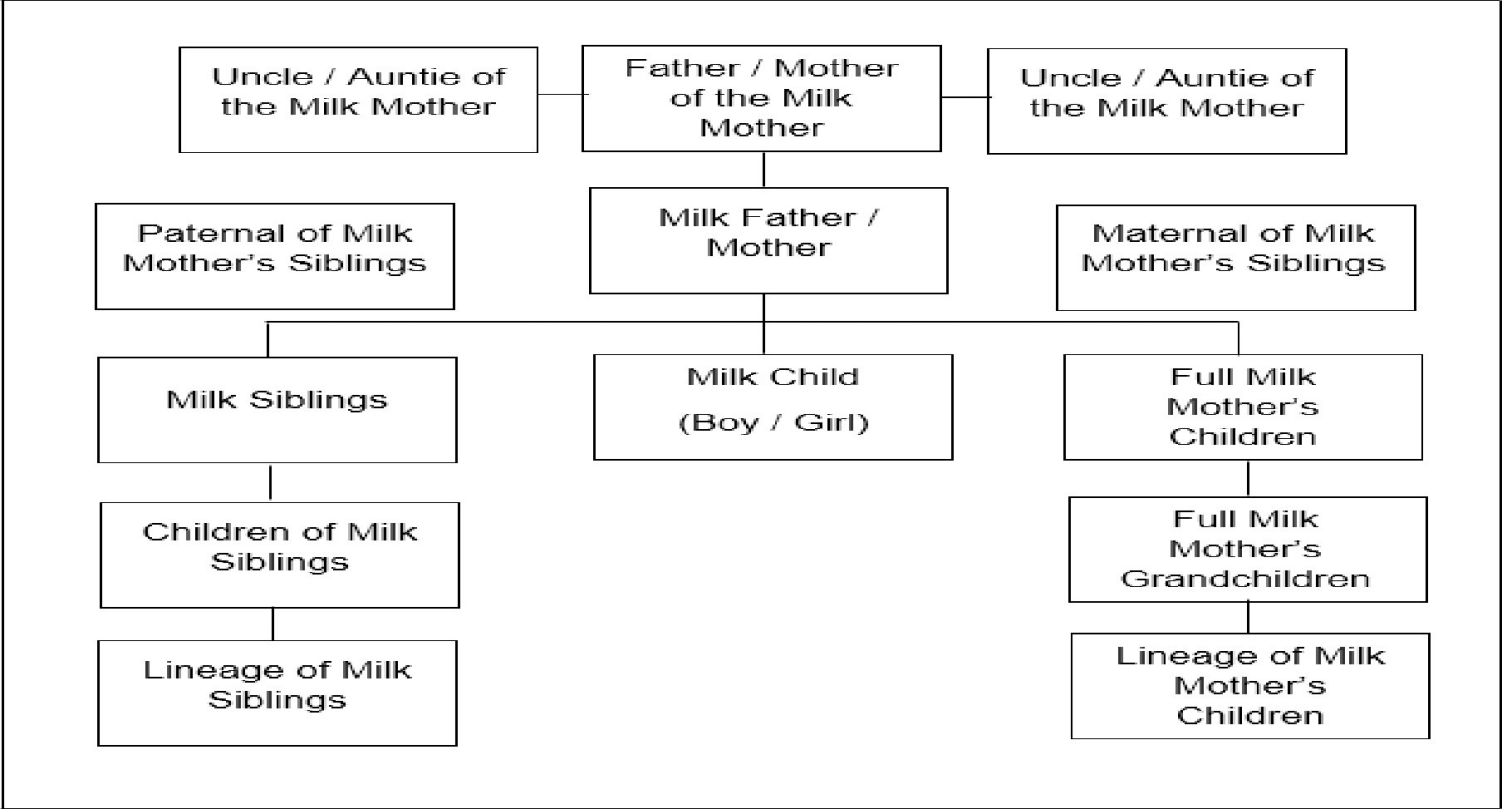
Relations forbidden because of milk ties.

The law on wet nursing is related to *mahram* (unmarriageability). According to Islamic legal terminology, mahram is a person who is forbidden to marry with a woman due to the existence of a kinship (descent), marriage or sisterhood relationship [19, 20]. Along with some conditions, a child fed on another woman’s milk becomes her milk child permanently. The milk child is strictly forbidden to marry the milk mother or milk father and their offspring or descendants due to their blood ties. Their children are also mahram (unmarriageable), and their milk kinship does not nullify ablution as they are allowed to mix as milk siblings, subject to aurat limits (or hijab rules), just as the milk child would with his/her own biological siblings. The lineage of the milk mother whom the milk child is forbidden to marry are her siblings, her sons and daughters, his/her female grandchildren and the mother of the milk mother. Likewise, the lineage of the milk father to whom the milk child is mahram (unmarriageable) include the milk father’s siblings; his/her daughters, even those from another wife; his/her female grandchildren and his/her mother. This is the consensus of the scholars of Syafi’i, Hanafi, Maliki and Hanbali [21]. The above discussion can be summarized in Fig 1.

In addition, the law describes the conditions for wet nursing that need to be understood to legally establish an infant’s status as a milk child. The conditions that are required to establish milk kinship are related to the woman’s breast milk, the amount of feeding, the age of the infant while feeding and the feeding mode [22]. All conditions stated above include that the milk mother is a woman who has reached puberty.

Regarding the amount of breastfeeding, according to Shafi’i School of Law, infants need to be satiated five or more times to establish milk kinship between the woman and the infant [23, 24]. In terms of the age of the infant, the majority of jurists agree that the milk child shall not be more than two years old for the law on mahram (unmarriageability) based on milk kinship to apply to both parties [25, 26].

In addition, the feeding mode also needs to be examined in order to determine the legal status of milk kinship. A consensus of scholars agree that an infant becomes a milk child if the infant is fed breast milk five or more times by the milk mother, regardless of whether the child is directly fed from the breast or using relevant medium such as a bottle, spoon, tube or syringe. Muslim society also needs to know the laws on feeding breast milk to someone else’s child in matters of guardianship, lineage and inheritance. In terms of lineage, a milk child does not take the milk father’s lineage. Likewise, the milk father is not its legal guardian [27]. In terms of inheritance, the milk child does not inherit from the milk mother nor she from him [28]. This discussion clearly shows that the legal status of milk kinship is an important matter that needs to be thoroughly understood by all parties involved, including the milk mother, the milk child and their respective families. Therefore, this study is significant in the context of the Muslim community so that milk mothers know the legal implication of wet nursing to prevent marriage among breastfeeding siblings in order to protect the sacred lineage. This study is also important to assist the authorities such as the Selangor Islamic Religious Council (MAIS) to enforce the betterment of wet nursing practices through legal approaches, policies and documentation of breastfeeding practices.

The practice of wet nursing has received tremendous attention among mothers in Malaysia, due to their awareness of the advantages of mother’s milk [29], as promoted by the government since 1992. For instance, the government introduced the “Baby-Friendly Hospital Initiative” as an effort to encourage the practice of suckling newborn babies. Furthermore, specific zones have been created for the purpose of suckling babies [30]. There is no specific control on the practice of wet nursing among Muslim society due to the federalism system in Malaysia that allocates religious matters to the state government’s jurisdiction (Federal Constitution of Malaysia). Most of the rulings on wet nursing such as the ruling that prohibits the activity of selling mother’s milk to others in order to prevent the uncertainty of the lineage among the Muslim community (Irsyad Fatwa of Federal Mufti office) and the ruling that prohibits milk banks in Malaysia [31] are given by Islamic religious councils. At the state level, the State of Selangor Islamic Religious Department introduced the ‘MyRadhaah’ card on 19 Nov 2018, which aims to record the practice of wet nursing among Muslims in that particular state [32].

## Literature review

Generally, countless research studies on milk children have been previously conducted by many researchers in Malaysia and international including Coutsoudis [33], Dusa et al. [7], Yusuf [34], Dennis [3], Ahluwalia et al. [35], Radzniwan et al. [36], Daud et al. [37], Ismail et al. [38], Mathur and Dhingra [39], Daud and Kusrin [40], Daud et al. [41], Azad et al. [42], and many others. Previous research mostly studied mothers’ understanding of the concept of wet nursing [39, 41], their understanding of breastfeeding exclusively [7], the attitude of milk mothers and their practice [41], the duration of their practice [3], the milk mothers’ rights and duties in feeding [40], the challenges experienced by career mothers [38] and the factors for the discontinuation of breast feeding by some mothers [35].

Some previous research studies also focused on the benefits of breast milk to an infant’s growth and health, such as by Kamaruddin et al. [43], Daud and Fatimah [29], Ward et al. [44], Rehman et al. [1], Berlanga-Macias et al. [2], Del Ciampo and Del Ciampo [45], Spiro [46], Gertosio et al. [4], Deborah et al. [5] and many more who empirically proved that mother’s milk is the best for infants. In addition, research on breastfeeding someone else’s infant were also conducted by Flores-Antón et al. [47]; Lommen et al. [48]; Saari [49], Mohamad [50] and Saari and Yusof [51], but these studies are more focused on breastfeeding others’ infants as adopted or foster children. Research on breastfeeding adopted or foster children was also conducted in the West by Gribble [52], who explained that breastfeeding is beneficial to mothers and children in terms of bonding and a calming effect.

Furthermore, there were also some research studies that focused on the laws on wet nursing in Malaysia, including the research performed by Dimon et al. [28] on Muslim society’s awareness of the laws related to wet nursing. They selected Muslim respondents from both genders to participate in their study. Contrarily, this research paper focused only on milk mothers who have engaged in wet nursing. In short, past research did not study wet nursing, especially milk mothers’ understanding of the legal implications of feeding someone else’s infant and the factors that encourage their willingness to feed them. Hence, this research is for the purpose of analyzing Muslim milk mothers’ understanding of the law and the legal implications of feeding breast milk to someone else’s infant and identifying the motivating factors for their willingness to do so.

## Material and methods

### Study design and setting

This study used a quantitative research design based on a purposive sampling method applied to 100 milk mothers aged 21 to 50 years old. Individuals who have consented to participate and had rich information on wet nursing were the targets group since the authors hoped that these individuals could provide more extensive data. The locations of this study included all nine districts (Sabak Bernam, Hulu Selangor, Kuala Selangor, Kuala Langat, Sepang, Hulu Langat, Gombak, Petaling and Klang) in Selangor, Malaysia. The names of the respondents were gathered from the Islamic Religious Department of Selangor. All respondents lived in Selangor covering nine districts namely Sabak Bernam, Hulu Selangor, Kuala Selangor, Kuala Langat, Sepang, Hulu Langat, Gombak, Petaling and Klang. Of these nine constituencies, most of the respondents live in the Petaling district of 28.0%. Meanwhile, Hulu Langat was the second-largest occupancy of 26.0%. This study also showed that there are two districts (Sabak Bernam and Hulu Selangor) that do not have the respondents. They were not cooperated to take part in the study. This study was conducted from January 2019 to July 2019.

### Sample and recruitment

The researchers selected the respondents through the information obtained from the authority, namely, the Selangor Islamic Religious Department. This research is only applicable to mothers who live in the state of Selangor, which was chosen as representative of the states in Malaysia. They were invited to participate in the study voluntarily. Those who need and wish to participate in this study must have provided written consent by signing the respondent consent form attached. The inclusion criteria of respondents were i) Mothers who breastfeed other children (at least one child), ii) Mother resident in Selangor, iii) Mother aged below 50 years old, iv) Literate and tech-savvy, and v) Consented to participate. Mothers under the age of 50 were chosen because mothers who breastfed their infants were revealed to be of reproductive age. The reproductive age group is typically described as 15–50 years old for demographic purposes. The exclusion criteria were the following: i) Non-Muslim respondents and ii) Muslims outside of Selangor or living in other Malaysian states.

### Data collection

The study was ethically approved by the Medical Research and Ethics Committee, Ministry of Health Malaysia with code NMRR-20-2503-57029 (IIR). Informed consent was acquired before each respondent was administered the questionnaire online. Before entering the study, all potential respondents were introduced to the study purpose and contents. After a comprehensive explanation of the study and acknowledgement of the anonymity and delinkage principle together with the other ethical considerations by the respondents, the respondents proceeded to answer the questions online based on their own willingness. This was implied consent—respondents who click on ‘I agree’ and complete the survey were deemed to have consented to participate.

The researchers developed a questionnaire survey to determine the practice and processes implemented by Muslim mothers. The online questionnaire was sent via e-mail. Respondents were contacted to get their email address so that questions can be emailed to them. This questionnaire was available in Malay language because most of the respondents were Malays, and they understood and preferred the language. On the first page of the questionnaire, the research details and the consent form were included. Respondents were given the option of completing the consent form in either English or Malay, and it was made clear that their participation was voluntary with no pressure to participate. This questionnaire took less than 30 minutes to complete. The data collection duration was 5 months after each respondent received the link. After 2 months, the link was disposed of and no other individual except the researcher could access it. This study did not present any risk to the respondents. However, if there were any sensitive matters that caused a respondent’s emotions to be disturbed while answering the questionnaire, the respondent could leave the website and the data that were entered were not be used or stored by the researcher.

Respondents’ personal information was kept confidential by the researcher. It will not be disclosed publicly unless required by law. The original personal information of the respondents may be viewed by the researcher, the ethics board of the institution of the researcher of this study and the regulatory authorities to verify the procedures and/or data of the clinical study. Respondent information will be stored in a computer for 2 years (24 months) and will be deleted from the computer after going through the analysis process.

The questionnaire consisted of two parts: sociodemographic characteristics and women’s wet nursing practices. The questionnaire in this research used a Likert scale to assess the understanding of the selected details on wet nursing. Likert scales allow respondents to easily operationalize personality traits or perceptions [53].

### Pilot study

A pilot study on 29 wet nursing Muslim mothers was conducted. The respondents asked to complete the questionnaire and provide feedback on the structure and nature of the questions. E-mail was used to deliver the online questionnaire. This pilot study was essential for assessing the validity of the instrument and evaluating the level of difficulty of the questions posed. The estimated time spent by each respondent for the entire questionnaire was recorded. In addition, the other aspects that might interfere with the data collection and analysis process were also identified. This pilot study is also designed to examine the reliability (internal consistency) of the instrument using Cronbach’s alpha. Cronbach’s alpha was used to assess the homogeneity of the questions to determine the internal consistency of the test. The researchers checked the Corrected Item Total Correlation (CITC) and compared the Cronbach’s alphas if any item was deleted. The overall value of the Cronbach’s alpha assessing the instruments of the pilot study exceeded the reference value (α = 0.8).

### Data analysis

The results for each questionnaire were compiled in Survey Monkey, an online survey program; and the total responses to each question were compiled for analysis. The data from this questionnaire were analyzed using the Statistical Package for Social Sciences (SPSS) version 22. Categorical variables were summarized as numbers and percentages whereas normally distributed continuous variables were presented as the means and standard deviations. A *p-value* less than 0.05 was considered statistically significant.

## Results

### Respondents’ characteristics

Table 1 shows that all respondents were wet nurses who lived in Selangor. The variables of the study were age, marital status, the number of children breastfed, residence, education level, occupation, and total income. The respondents in this study were 21 to 50 years old. The results showed that all respondents were married (100.0%). In terms of education, the respondents were examined according to various educational backgrounds: *Sijil Pelajaran Malaysia* (SPM), certificate, diploma, bachelor’s degree, master’s degree, and the highest level of doctor of philosophy. The study showed that 48.0% of respondents had a bachelor’s degree, which outnumbered respondents from other education levels. In terms of employment, 37.0% of the respondents were civil servants, 32.0% of the respondents worked in the private sector, and only 3.0% of the respondents were students. Therefore, most of the respondents in this study are professionals who have a monthly income that ranged from USD717 to USD1432 (40.0%). In addition, 31.0% of the respondents had a monthly income that ranged from USD238 to USD716, and only 17.0% of respondents had a monthly income below USD238.

**Table 1:** Sociodemographic characteristics of respondents.

Item	Respondents	
	Frequency (n)	Percentage (%)
**Age (years)**		
21–30	30	30.0
31–40	64	64.0
41–50	6	6.0
**Marriage status**		
Married	100	100.0
**Residence (district)**		
Kuala Selangor	3	3.0
Kuala Langat	5	5.0
Sepang	8	8.0
Hulu Langat	26	26.0
Gombak	12	12.0
Petaling	28	28.0
Klang	18	18.0
Sabak Bernam	0	0
Hulu Selangor	0	0
**Education level**		
SPM	7	7.0
Certificate	2	2.0
Diploma	20	20.0
Bachelor	48	48.0
Master	19	19.0
PhD	4	4.0
**Occupation**		
Civil sector	37	37.0
Private sector	32	32.0
Self-employed	13	13.0
Housewife	15	15.0
Student	3	3.0
**Income**		
Below USD238	17	17.0
USD238-USD716	31	31.0
USD717-USD1432	40	40.0
USD1433-USD2148	10	10.0
USD2149- above	2	2.0

### Understanding of wet nursing in Islamic law

The research findings addressed milk mothers’ understanding of the law on feeding breast milk to someone else’s infant or wet nursing (Table 2) and the factors for their willingness to do so (Table 3). This research finds that the item with the highest mean value is ‘A milk child is forbidden to marry his milk mother or milk father’ (mean = 3.67), followed by the item ‘A milk daughter/son is forbidden to marry the biological sons and daughters of his milk mother’ (mean = 3.65), the item ‘Someone else’s infant aged not more than two years old and fed with the breast milk of a woman can be her milk infant’ (mean = 3.57), the item ‘Islam permits feeding an infant with expressed milk’ (mean = 3.55), and the item ‘A mother may feed her milk to someone else’s infant subject to her husband’s permission’ (mean = 3.52).

**Table 2:** Understanding of wet nursing in Islamic law.

No	Item	Strongly Disagree	Dis-agree	Agree	Strongly Agree	Mean±SD
1	It is forbidden for a milk child to marry his milk mother/father.	1	0	30	69	3.67±0.53
2	A milk daughter/son is forbidden to marry the biological child of her/his milk mother.	1	0	32	67	3.65±0.54
3	Someone else’s infant aged not more than two years old and fed with the breast milk of a woman can be her milk child.	0	4	35	61	3.57±0.57
4	Islam permits feeding an infant with the expressed breast milk of the milk mother.	0	4	37	59	3.55±0.58
5	A mother may feed her milk to someone else’s infant subject to her husband’s permission.	1	1	43	55	3.52±0.58
6	The *aurat* limits (or *hijab*) for a milk child in relation to his milk parents are the same as those for his biological parents.	0	4	49	47	3.43±0.57
7	To develop milk kinship between the mother and the child, the child must be satiated at least five times through feeding.	2	10	34	54	3.40±0.75
8	Ablution is nullified if a milk child touches his milk parents.	4	9	40	47	3.30±0.80
9	A milk child takes the lineage of his/her biological parents.	3	14	47	36	3.16±0.78
10	A milk father can be the guardian for a milk daughter’s marriage.	41	31	20	8	1.95±0.97
11	A milk child may inherit the property of his milk parents.	38	43	17	2	1.83±0.78
12	Islam allows a milk mother to feed someone else’s infant only through breastfeeding.	50	47	2	1	1.54±0.59

**Table 3:** Motivating factors that influence milk mothers to breastfeed.

No.	Item	Strongly Disagree	Disagree	Agree	Strongly Agree	Mean±SD
1	I feed my breast milk to someone else’s infant because I have surplus milk.	4	12	46	38	3.18±0.80
2	I feed my breast milk to some else’s infant as a source of income.	60	39	1	0	1.41±0.51
3	I feed my breast milk to someone else’s infant to help mothers who do not have enough breast milk.	2	6	46	46	3.36±0.69
4	I feed my breast milk to someone else’s infant because its mother has health problems that prevent breastfeeding.	5	22	40	33	3.01±0.87

This research finds that the item ‘The aurat limits (or hijab) for a milk child in relation to his milk parents are the same as for his/her biological parents’ scored a moderately high mean (mean = 3.43); and this item was followed by the item ‘The legal condition which establishes the status of milk mother is five different feedings times until satiety’ (mean = 3.40), the item ‘Ablution is nullified if a milk child touches his milk mother/father’ (mean = 3.30) and the item ‘A milk child takes the lineage of his biological parents’ (mean = 3.16). Research results find that the items with the lowest mean values are the item ‘A milk father can be the guardian of his milk daughter’s marriage’ (mean = 1.95), the item ‘A milk child can inherit property from his milk parents’(mean = 1.83) and ‘Islam permits milk mothers to feed someone else’s infant only through breastfeeding’ (mean = 1.54).

The analysis using SPSS related to the above findings is summarized in Table 4 shows that 100% of respondents have a high level of understanding related to the legal implications (Islamic law) of wet nursing.

**Table 4:** Level of understanding of wet nursing in Islamic law.

Level	Frequency	Percentage (%)
0–12 (Low)	0	0
13–25 (Medium)	0	0
26–48 (High)	100	100
Total	100	100.0

### Motivating factor influencing the wet nursing practice

Based on Table 3, the reason that milk mothers breastfeed with the highest mean is ‘I feed my milk to someone else’s infant to help mothers who do not have enough breast milk’ (mean = 3.36). This is followed by the item ‘I feed someone else’s infant because I have surplus breast milk’ (mean = 3.18) and the item ‘I feed someone else’s infant because its mother has a health problem that prevents breastfeeding’ (mean = 3.01). The item that obtained the lowest mean is ‘I feed someone else’s infant as a source of income’ (mean = 1.41).

### Payment received during wet nursing

As Table 5 shows, the majority of respondents did not receive any payment or charged any fees for wet nursing (94.0%). However, some respondents received a payment of RM100/USD24(4.0%), RM1/USD0.2 (1.0%) and RM50/USD12 (1.0%) for wet nursing. This clearly shows that almost all respondents voluntarily breastfed other children at no charge.

**Table 5:** Total payment received during wet nursing.

No	Total payment (RM)	Frequency (n)	Percentage (%)
1	No charge	94	94.0
2	RM1/USD 0.2	1	1.0
3	RM50/USD12	1	1.0
4	RM100/USD24	4	4.0

## Discussion

This research found that 99.0% of respondents agree that a milk child is forbidden to marry his milk mother or milk father. Only 1.0% disagrees with this item. This showed that only one person among the respondents did not understand the permanent prohibition of marriage between a milk child and his milk mother or father. These findings showed that the respondents understood the legal implications of wet nursing because of blood ties, as mentioned in the introduction. The findings are further strengthened by the results of the analysis of the level of understanding, which found that 100% of respondents have a high level of understanding related to the legal implications of wet nursing. This research findings are in line with a finding by Dimon et al. [28] that 87.0% of respondents agree that a milk son is prohibited from marrying his milk mother and 71.5% of respondents agree that a milk daughter cannot marry her milk mother’s husband.

The item that scored the second highest mean value is focused on marriage law, namely, ‘A milk daughter/son is forbidden to marry the biological sons and daughters of his milk mother’ (mean = 3.65). Approximately 67.0% of respondents in this research strongly agree and 32.0% agree with this item statement. Only 1.0% strongly disagrees with it. This finding also showed that only one respondent does not understand the legal permanent prohibition of marriage between a milk child and the biological offspring of its milk mother. Generally, this research found that milk mothers understand the legal implications of feeding breast milk in terms of marriage laws, namely, it is forbidden for a milk child to marry their milk mother or father and milk siblings just as he/she is forbidden to marry his own biological mother or father and his biological siblings by the same parents or the same mother or the same father [24, 25, 28]. In addition, the item that scored the third highest mean relates to the age of the infant fed as a milk child: ‘Someone else’s child aged not more than two years old and fed with breast milk can be a milk child’ (mean = 3.57). Here, 96.0% of respondents agree that only an infant aged not more than two years and fed with the breast milk of the milk mother becomes a milk child. Generally, this research found that respondents understand the legal age of the fed infant required to establish milk kinship [25, 54–56].

In addition, research respondents also well understood the legal condition of 5 different feeding times until satiety in the item ‘, ‘to develop milk kinship between the mother and the child, the child must be satiated at least five times through feeding (mean = 3.40) with 88.0% agreement. This is in line with Muslim scholars’ views [23–25, 55, 57]. Research respondents also well understood the modes of feeding breast milk to establish milk kinship in the item ‘Islam permits feeding an infant with expressed milk of milk mother’ (mean = 3.55). This proved that respondents well understood that besides breastfeeding, milk can also be fed through other modes such as expressed milk in a bottle [22, 56]. In addition, the item ‘A mother can feed her milk to someone else’s infant subject to her husband’s permission’ received 98% agreement among respondents (mean = 3.52) and was fifth highest in this research. This showed that respondents understood that a husband’s permission is essential before making a decision to feed breastmilk to someone else’s infant [24, 58]. In addition, 72.0% of research respondents do not agree that the milk father can be the guardian in a milk daughter’s marriage while only 28.0% agree. The basic law regarding this matter is that a milk father cannot be the guardian of his milk daughter in her marriage. This showed a deficit in the understanding of milk mothers regarding this matter [24, 27, 59].

This research also found that 87.0% of respondents agree (mean = 3.30) with the item ‘Ablution is nullified if a milk child touches his milk parents’. This shows there is still a deficit in understanding because ablution is not legally cancelled by skin contact between milk child and milk parents. 96.0% of respondents agree (mean = 3.43) with item ‘The aurat limits (or hijab) of a milk child to his milk parents are the same as to his/her biological parents’. This finding is in line with Shariah law and shows that respondents have a good understanding of aurat limits (or hijab). In addition, some respondents lack the understanding that a milk child takes the lineage (*nasab*) of his biological parents, as shown by 17.0% of respondents who disagree with this item. There is also a deficit in the respondents’ understanding concerning inheritance whereby 19.0% of the respondents agree that a milk child may inherit the property of his milk parents.

In Shariah law, ablution is not nullified by touching between a milk child and his milk parents, the aurat limits (or hijab) for the milk child are the same for its milk parents as for its biological parents and skin contact is permissible between milk children and milk parents. In terms of lineage (nasab), a milk child does not take the lineage of his milk parents. Likewise, a milk father is not the legal guardian of his milk child. Similarly, regarding mahram (unmarriageability), a milk child is forbidden to marry his milk parents and milk siblings [25, 27]. Finally, regarding inheritance, a milk child does not inherit from the milk mother and neither does she from her milk son/daughter [24, 28, 59].

These findings showed certain aspects of the legal implications of milk kinship that are still not well understood by some milk mothers. These findings are in line with research findings by Musa et al. [60] and Zilal [49] that there is an issue of some milk mothers still not fully understanding the legal implications of milk kinship. These findings strengthen the view that milk mothers need to improve or enhance their understanding of the legal implications of milk kinship.

The research also found that respondents engage in wet nursing to help mothers who do not have sufficient milk and sympathized with the infants whose mothers face health problems that prevent them from breastfeeding their infants. This practice is a good deed as the intention is to help others in need. It is consistent with Islamic teachings as al-Quran states ‘Help you one another in righteousness and piety,’ (al-Quran, al-Maidah 5:2) and ‘So race to (all that is) good’ (al-Quran, al-Baqarah 2:148). The majority of research respondents, namely, 84%, feed someone else’s infant because they have surplus milk (mean = 3.18). This is also a good practice in not letting surplus milk go to waste and sharing a sustenance of abundant or excessive milk bestowed by feeding the surplus to someone else’s child.

Generally, wet nursing is in line with Islamic teachings on the basis of mutual help. This awareness among Muslim women has encouraged them to help each other and encouraged mothers to provide breast milk instead of formula milk, which is very nourishing for the physical and mental health of babies and very young children [39, 43]. Furthermore, this study found that the item ‘I feed someone else’s infant as a source of income’ (mean = 1.41) has received the least agreement from respondents. This finding shows that research respondents are more inclined to do good to help fellow Muslims by helping other mothers in feeding their infants with breast milk. Only 1% of respondents practice wet nursing as a source of income.

### Strengths and limitations of the study

Some strengths of this study include providing novel information regarding wet nursing, influences, and practices among a group of Muslim mothers living in Selangor. This study also utilized primary data that should also be considered as a strength of this study. However, there can be recall bias among the respondents since the information was collected retrospectively one to two years after wet nursing was completed. Therefore, we cannot rule out recall bias in the responses given in the interviews. In this sense, it is possible that the reported breastfeeding rates overestimated the actual rates.

## Conclusion

This study found that the majority of respondents understand the laws relating to feeding breast milk to someone else’s infant, especially laws involving marriage and conditions for and modes of feeding breast milk. However, respondents still do not fully understand the laws relating to mahram (unmarriageability), *nasab* (lineage), guardianship and inheritance involving a milk son/daughter. The research findings show there are quite a variety of factors for women to become a milk mother including having surplus breast milk, helping mothers who are not able to breastfeed due to underlying illnesses and as a source of income. This research is hoped to create awareness among people who are linked by milk kinship and Muslim society on the importance of understanding the Shariah laws involving feeding breast milk to someone else’s infant in order to protect the sacred lineage and the well-being of Muslim families. The findings of this study have been presented to the Selangor Islamic Religious Council (MAIS) to emphasize the need to enact specific legal provisions in the Selangor Islamic Family Law Enactment 2003 in order to regulate the practice of wet nursing among Muslims in Selangor. Finally, this study suggests that further research be conducted on the challenges and implementation methods of developing a data base to record the wet nursing practices in Selangor and Malaysia in order to maintain the sacred lineage and the happiness of the Muslim family.

## Supporting information

S1 Questionnaire (PDF)

S1 Data (XLSX)

## Abbreviations

CITC: Corrected Item Total Correlation
SPSS: Statistical Package for Social Sciences
SPM: *Sijil Pelajaran Malaysia*, and PhD: Doctor of Philosophy
