# Supplementary material for: A quantitative study on Muslim milk mother’s understanding of the Islamic concept of wet nursing
Source: PLoS One. 2022 May 19;17(5):e0265592. doi: 10.1371/journal.pone.0265592 (PMC9119450; doi:10.1371/journal.pone.0265592)
Supplement: S1 Questionnaire — (PDF) [file pone.0265592.s001.pdf]

Serial No.: _________

QUESTIONNAIRE
THE PRACTICE OF WET NURSING (RADHA'AH) AMONG THE MUSLIM COMMUNITY IN THE STATE OF SELANGOR

Purpose of Study

To all the respected wet nursing mothers in the state of Selangor, the Selangor Islamic Religious Council (MAIS) has given us (the researchers) the responsibility to conduct research to find out more about the practice of wet-nursing other people's children (Radha'ah) in the Muslim community in the State of Selangor. Therefore, we seriously need the co-operation of wet nursing mothers for the purpose of collecting data to complete this research. All information is confidential and is used for research purposes only. We request the cooperation of wet nursing mothers to answer this questionnaire.

Respondents who have completed the questionnaire will be contacted for giving of a small token sum of RM10 as consolation money.

Head Researcher
Assoc. Prof. Dr. Nurhidayah Binti Muhammad Hashim (UiTM)
puan.hidayah2@gmail.com

Research Members:
Assoc. Prof. Dr Salasiah Binti Hanin Hamjah (UKM)
Assoc. Prof. Dr Latifah Binti Abdul Majid (UKM)
Assoc. Prof. Dr Zuliza Binti Kusrin (UKM)
Assoc. Prof. Rafeah @ Rapengah Binti Saidon (UiTM)
Dr. Nora'inan Binti Bahari (KUIS)
Dr. Norsyamlina Binti Che Abdul Rahim (KKM)

THIS QUESTIONNAIRE CONSISTS OF 3 PARTS:

- **Section A**: Personal Information
- **Section B**: Understanding
  B: Understanding Rulings Regarding Breastfeeding Other People's Children
- **Section C**: Practices
  C: Practices Regarding Breastfeeding Other People's Children (wet nursing)

All your co-operation is greatly appreciated and we thank you very much.

SECTION A: PERSONAL INFORMATION

Instruction: Please tick (√) the appropiate answer

1. Age
( ) 18 Years Old And Below
( ) 21-30 Years Old
( ) 31-40 Years Old
( ) 41-50 Years Old
( ) 50 Years Old And Above

2. Phone Number :
____

3. Marriage Status
( ) Single
( ) Married
( ) Divorcee/Widow

4. Number of children breastfed

Specify: ____________________

5. Residence (District)
( ) Sabak Bernam
( ) Hulu Selangor
( ) Kuala Selangor
( ) Kuala Langat
( ) Sepang
( ) Hulu Langat
( ) Gombak
( ) Petaling
( ) Klang

5. Highest Level of Education
( ) SPM
( ) Certificate
( ) Diploma
( ) Bachelor's Degree
( ) Master's Degree
( ) Doctor of Philosophy

6. Occupation
( ) Civil Servant
( ) Private Sector
( ) Self-employed
( ) Housewife
( ) Student

7. Source of Information on the Rulings of Breastfeeding are obtained from:
( ) Print Media
( ) Electronic Media
( ) Formal Education
( ) Non-Formal Education
( ) Others : (Specify):_____________

8. Income
( ) Below RM1000
( ) RM1000-RM3000
( ) RM3001-RM6000
( ) RM6001-RM9000
( ) RM9001- and Above

SECTION B: UNDERSTANDING

Please circle the appropiate answer based on the scale below:

Strongly Disagree	Disagree	Agree	Strongly Agree
1	2	3	4

B : UNDERSTANDING OF RULINGS REGARDING BREASTFEEDING OTHER PEOPLE'S CHILDREN

B1	Wet nursing is allowed or permissible according to Islamic law.	1	2	3	4
B2	A milk father can be the guardian for a milk daughter's marriage.	1	2	3	4
B3	Someone else's infant aged not more than two years old and fed with the breast milk of a woman can be her milk child.	1	2	3	4
B4	To develop milk kinship between the mother and the child, the child must be satiated at least five times through feeding.	1	2	3	4
B5	Mothers with dangerous diseases such as HIV, TB, Cancer, and Hepatitis must not breastfeed.	1	2	3	4
B6	Islam permits feeding an infant with the expressed breast milk of the milk mother.	1	2	3	4
B7	Breastfeeding a wet nurse's milk becomes obligatory (wajib) if an infant is allergic to any food and drink.	1	2	3	4
B8	A husband is obliged to provide for his wife who is wet nursing another person's infant	1	2	3	4
B9	A husband has the right to prohibit his wife from wet nursing other people's infants.	1	2	3	4
B10	A husband has the right to prohibit his wife from taking wages for wet nursing other people's infants.	1	2	3	4
B11	A wet nurse can get wages from the biological parents of the breastfed infant.	1	2	3	4
B12	A milk child takes the lineage of his/her biological parents.	1	2	3	4
B13	A mother may feed her milk to someone else's infant subject to her husband's permission.	1	2	3	4
B14	It is forbidden for a milk child to marry his milk mother/father.	1	2	3	4
B15	A milk daughter/son is forbidden to marry the biological child of her/his milk mother.	1	2	3	4
B16	A milk daughter can perform Umrah and Hajj together with her milk father or milk brother (as her mahram)	1	2	3	4

B17	The *aurat* limits (or *hijab*) for a milk child in relation to his milk parents are the same as those for his biological parents.	1	2	3	4
B18	A milk child may inherit the property of his milk parents.	1	2	3	4
B19	Islam allows a milk mother to feed someone else's infant only through breastfeeding.	1	2	3	4
B20	An infant who is fed breast milk using a bottle does not qualify as a milk child.	1	2	3	4
B21	Breast milk can be sold for the use of other people's infants.	1	2	3	4
B22	A woman can take medications to stimulate the production of breast milk to wet nurse other people's infants.	1	2	3	4
B23	It is permissible for a woman's infant to breastfeed from her husband's sister (her sister-in-law), who then becomes the infant's milk mother (wet nurse).	1	2	3	4
B24	Infants who drink breast milk mixed with other foods or beverages can qualify as milk children.	1	2	3	4
B25	Milk that is frozen into cheese or thawed, if given to a baby will turn it into a milk child.	1	2	3	4
B27	Ablution is nullified if a milk child touches his milk parents.	1	2	3	4

SECTION C: PRACTICES

Please tick (√) the appropiate answer

C1. THE PRACTICE OF WET NURSING OTHER PEOPLE'S INFANTS

C1	I feed my breast milk to someone else's infant because I have surplus milk.	1	2	3	4
C2	I feed my breast milk to some else's infant as a source of income.	1	2	3	4
C3	I feed my breast milk to someone else's infant to help mothers who do not have enough breast milk.	1	2	3	4
C4	I feed my breast milk to someone else's infant because its mother has health problems that prevent breastfeeding.	1	2	3	4

C2. Experience / Wet nursing challenges

1	I have experienced forgetting whose child I had wet nursed.	1	2	3	4
2	I have witnessed a situation where a milk child is in a relationship with a wet nurse's immediate family member (milk kinship).	1	2	3	4
3	I have experienced not being allowed to perform umrah with my milk son.	1	2	3	4
4	I have witnessed a situation where a marriage is dissolved because a milk child was married to the wet nurse's family member (milk kinship).	1	2	3	4
5	I once faced a situation where a milk child experienced emotional stress after knowing his/her position.	1	2	3	4
6	I have witnessed a misunderstanding between a biological child and a milk child (milk kinship).	1	2	3	4
7	I have witnessed a close relationship between a biological child and a milk child (milk kinship).	1	2	3	4
8	I have witnessed a wet nurse's husband becoming a marriage guardian (wali) for a milk child.	1	2	3	4
9	I had gifted (hiba) assets to my milk child.	1	2	3	4
10	I once bequeathed (by will) property to my milk child.	1	2	3	4

C3. If you have ever received payment for wet nursing, please state the amount: RM: _________________

C4. Frequency of wet nursing other people's infants? (tick only one)

No	Breastfed Child	Less than 5 times	More than 5 times
1	First Child		
2	Second Child		
3	Third Child		
4	Fourth Child		
5	Fifth Child		
6	Sixth Child		

7	If more, please specify		

C5. The method of wet nursing you practise (can tick more than one)

( ) Directly from the breast
( ) with a cup
( ) with a nursing bottle
( ) with a syringe
( ) with a tube (Supplemental Nursing System)
( ) Others: Specify:_______________________________

C6. Have you ever mixed milk with other foods? If so, please mark (√) on the statement below (can tick more than one)

( ) Breast milk mixed with formula milk (powdered)
( ) Breast milk mixed with cereal (e.g., Nestum)
( ) Breast milk mixed with porridge (e.g., rice)
( ) Breast milk mixed with biscuits
( ) Others: Specify:_______________________________

C7. Other experiences

No	Other Experiences	Yes	No
1	Do you recognize your milk child?		
2	Does your family know the identity of your milk child?		
3	Do you record the background information of your milk child?		
4	Do you feel the necessity to record the background of your milk child?		
5	Do you get the permission from your husband (if married) before wet-nursing?		
6	Do you still contact your milk child?		
7	Do you know the current location of your milk child?		
8	Do you get support from your family tonurse a child?		

8. In your opinion, how can we record more systematically about wet nursing or breastfeeding other people's infants?

……
……
……
……
……
……
……
……
……
……

- Thank you for your co-operation -
